# Late detection of cleft palate

**DOI:** 10.1007/s00431-015-2590-9

**Published:** 2015-08-01

**Authors:** K. H. Hanny, I. A. C. de Vries, S. J. Haverkamp, K. P. Q. Oomen, W. M. Penris, M. J. C. Eijkemans, M. Kon, A. B. Mink van der Molen, C. C. Breugem

**Affiliations:** Department of Paediatric Plastic Surgery, Wilhelmina Children’s Hospital, University Medical Centre, PO Box 85500, 3508 GA Utrecht, The Netherlands; Department of Speech and Language Therapy, Wilhelmina Children’s Hospital, University Medical Centre, Utrecht, The Netherlands; Department of Otolaryngology-Head and Neck Surgery, Wilhelmina Children’s Hospital, University Medical Centre, Utrecht, The Netherlands; Faculty of Behavioral and Social Sciences, University of Groningen, Groningen, The Netherlands; Department of Biostatistics and Research Support, Julius Centre, University Medical Centre, Utrecht, The Netherlands

**Keywords:** Cleft palate only, Diagnosis, Children, Age, Feeding difficulties, Nasal regurgitation

## Abstract

Cleft palate only (CPO) is a common congenital malformation, and most patients are diagnosed within the first weeks after birth. Late diagnosis of the cleft palate (CP) could initially result in feeding and growth impairment, and subsequently speech and hearing problems later in life. The purpose of this study is to retrospectively investigate (1) at which age CPO is diagnosed and (2) how the presence of syndromes and other factors relate to the age at diagnosis. The mean age of all children at our centre with CPO included between 1997 and 2014 at diagnosis (*n* = 271) was 1 year and 4 months. In all, 24.8 % (*n* = 67) was older than 12 months when diagnosed, and 37.3 % (*n* = 101) of all children had been diagnosed >30 days. These findings remain valid when a cut-off point of 14 days is used (44.3 % late). Moreover, the grade of the cleft was a determining factor for successful diagnosis; submucous clefts were detected much later on average (89.3 % > 30 days; *p* = .000). Similar results were found using Kaplan-Meier survival analyses.

*Conclusion*: CPO is often diagnosed late. Patients diagnosed ≤30 days after birth more often presented with an associated disorder. Early diagnoses became more frequent as the severity of the cleft increased (grades 1–4). Professionals should perform more thorough intra-oral investigations, including manual palpations and visual inspections of the palate; they should be made more aware of the frequent accompanying symptoms.

**Table:** 

**What is Known:** • *The presence of cleft palate only (CPO) is known to negatively affect feeding, hearing, speech and (social) development.* • *Submucous clefts are often underdiagnosed due to their difficulty to detect. As far as we know the literature shows that symptomatic submucous CPs are often diagnosed at an average age of 4.9 years.*
**What is New:** • *37.3 % respectively of all children with CPO were diagnosed relatively late (>30 days after birth), 24.8 % was older than 12 months when diagnosed. Mean age of all children with CPO was 1 year and 4 months.* • *We conclude that midwives and pediatricians should perform more through intra-oral investigations of all new-borns, including both a manual palpation, als well a visual inspection of the palate.*

## Introduction

Oral clefts (OC) [25] frequently encountered craniofacial malformations and are generically divided into three groups: cleft lip (CL), cleft lip with cleft palate (CL/P), and cleft palate only (CPO). Depending on geographical location and racial ethnicity, the worldwide incidence of clefts is 1 in 1000 births for CL/P and 1 in 2000 births for CPO [25]; Asian ethnicity shows a higher incidence compared to Caucasian ethnicity, and African ethnicity shows the lowest incidence [25].

When looking at geographical location, at 13.50 in 10,000 births, the prevalence of CL/P in the Netherlands is higher than in all other European counties, except for Germany (13.94 in 10,000 births). Moreover, at 5.07 in 10,000 births, the prevalence of CPO in the Netherlands is relatively low as compared to the rest of Europe, especially as compared to Finland, which shows the highest European prevalence at 14.31 in 10,000 births [26].

**Fig. 1:**
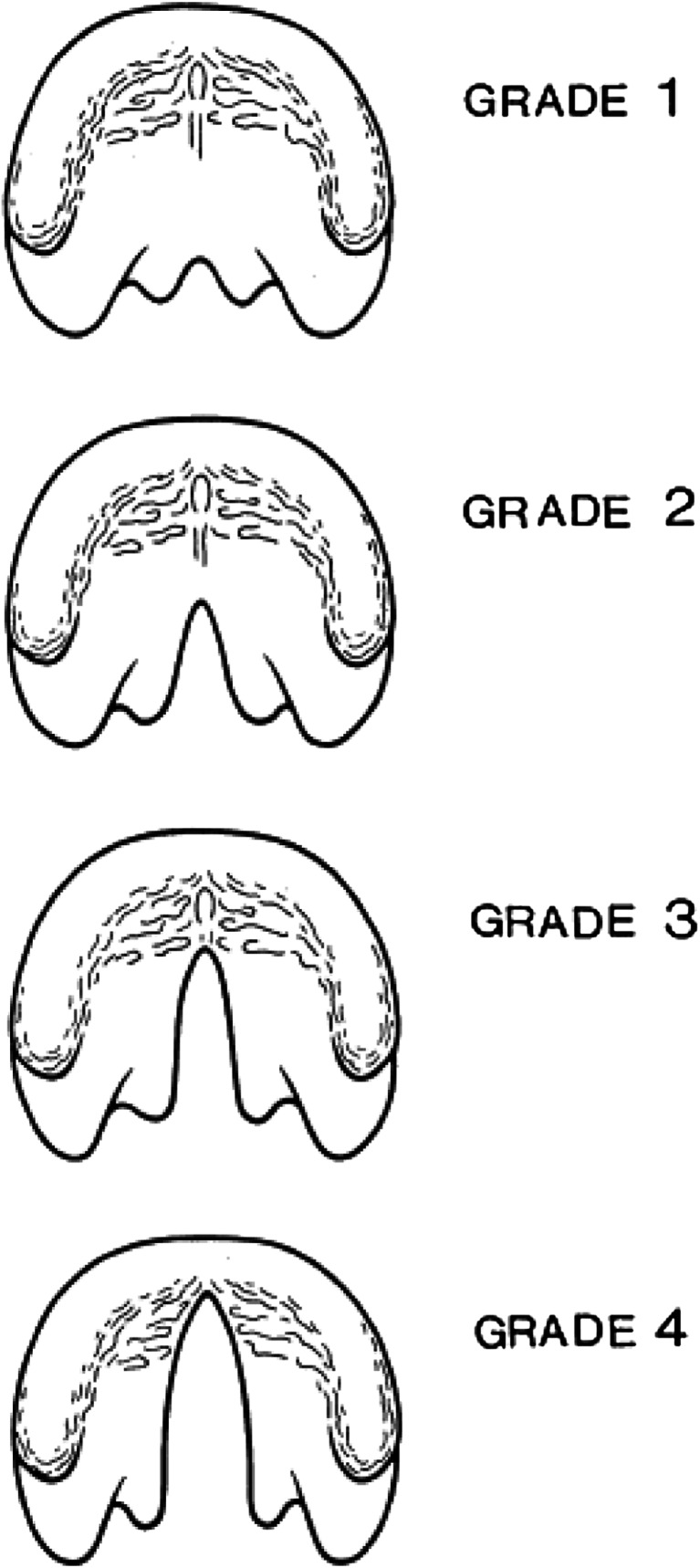
Grade of the cleft palate. For the present study, the grade of the cleft was divided into four grades (based on Jensen et al. 1988); *grade 1*, submucous cleft/bifid alveolus; *grade 2*, soft palate only; *grade 3*, soft palate and <1/3 hard palate; *grade 4*, complete CP

The extent of CPO can range from a small indentation in the uvula (bifid uvula) to a complete cleft of the soft and hard palate [17]. Clefts of the palate are generally categorised into four grades (Fig. 1): grade 1, submucous cleft/bifid uvula; grade 2, soft palate only; grade 3, soft palate and <1/3 hard palate; grade 4, complete CP [17]. The extent of the cleft increases with grade number [17].

The presence of CPO is known to negatively affect feeding, hearing and speech [6, 9]. Frequent early symptoms include feeding difficulties, nasal regurgitation, malnutrition, failure to thrive and hearing loss, while later in life, speech and voice problems and orthodontic problems often necessitate treatment [6, 7, 10, 14]. Consequently, early diagnosis of CPO is preferable, and raising awareness among health care professionals about (the early detection of) CPO remains important. It is hypothesised that submucous clefts are often underdiagnosed due to their detection difficulty. Maarse et al. underline the evidence that the different cleft categories are variously associated with additional congenital anomalies and underlying chromosomal defects. This emphasises that associated disorders are known to coincide with the cleft palate (CP) [19].

Although most oral clefts are diagnosed soon after birth [26], Reiter et al. have shown that a symptomatic submucous CP is often diagnosed at an average age of 4.9 years [20]. Since only submucous clefts were analyzed, no information about the age at detection for other grades of CP is present. The importance of an early diagnosis has been demonstrated by Chapman et al. and Jansonius-Schultheiss [4, 16], demonstrating that phonologic development is delayed due to CPO as a result of articulatory incompetence, as compared to normal children.

The purpose of this study is to retrospectively investigate (1) at which age CPO is diagnosed in a large tertiary children’s hospital and (2) how the presence of syndromes, the extent of the CP, gestational age (GA) and the location of delivery relate to the age at diagnosis and the reason for presentation.

## Methods

The data for this study were gathered by exploring the medical records of 271 consecutive patients with CPO, registered in the National Dutch Cleft Registry (NVSCA) between January 1997 and January 2014. All patients were treated by the cleft palate team of the Wilhelmina Children’s Hospital (WCH), Utrecht in the Netherlands. Approval of this study was obtained from the Medical Ethical Board.

In the Netherlands, regardless of age, all live-born patients with an OC are registered into the Dutch Cleft Registry (DCR) before reconstructive cleft surgery is performed [1, 18]. Registration is performed during the first visit to the cleft palate team (registration date). Although the Dutch prenatal guideline suggests that children born with a cleft should be seen by a cleft palate team within the first 2 weeks after birth, most children are seen within the first 2 to 4 weeks after birth. This 2-week period is sometimes extended to 3–4 weeks due to extraneous factors such as, e.g. parent illness, holidays, but rarely exceeds 4 weeks. The Dutch Cleft Registry has been systematically validated [22].

From this data set, all children with an OC other than CPO were excluded (e.g. CL, CL/P). Moreover, adopted children were excluded due to a lack in neonatal and maternal records.

The following demographic characteristics were analysed: gender, date of birth, date of registration, grade of CPO (grades 1–4 according to Jensen et al. [17]; Fig. 1), presence of syndromes and/or congenital anomalies, delivery location, GA and the age at diagnosis of CPO. Additionally, this final measure was also converted into two cut-off variables: AGE30, containing two groups: early (≤30 days) or late (>30 days) age at diagnosis; and AGE14, comprising two groups: early (≤14 days) or late (>14 days) age at diagnosis. Finally, GA was also converted into a dichotomous variable: pre-term (<37 weeks) and full-term (≥37 weeks).

Our definition of “CPO with an associated disorder” included children with CPO with one or more of the following: a syndrome, a congenital abnormality or a psychomotor retardation. In some cases, when a syndrome was strongly suspected but had not been diagnosed (yet), this was also defined as an associated disorder (CPO+).

These data were subsequently collected into a SPSS database [15]. Descriptive statistics were used to summarise information on infant and maternal characteristics, as well as information on syndromes and anomalies. The dichotomous age at diagnosis variables was compared with the continuous variables using an independent *t* test, and with the categorical variables using a chi-squared test. Additionally, the continuous age at diagnosis variable was compared with the other variables using a Kaplan-Meier survival analysis (censoring n/a; all patients were diagnosed) [21]. All descriptive data are presented as percentages and as means ± 1 standard deviation (SD). Where applicable, IQRs are also provided.

## Results

Of the 271 patients diagnosed, 59.0 % were female (*n* = 160), and 46.5 % (*n* = 126) had an associated disorder. Table 1 gives an overview of patient characteristics.

**Table 1:** Characteristics of all included children with CPO

Summary of characteristics of included children	
	CPO patients (*n* = 271)
Gender	
Male	41.0 % (*n* = 111)
Female	59.0 % (*n* = 160)
Syndrome and/or congenital abnormalities	
Yes	46.5 % (*n* = 126)
No	53.5 % (*n* = 145)
Gestational age (week)	
Pre-term (<37 weeks)	11.1 % (*n* = 30)
A term (>37 weeks)	81.5 % (*n* = 221)
Mean and SD	273.94 (39 weeks) SD 15.261 (2.1 weeks)

### Age at diagnosis (after birth)

Table 2 provides an overview of when diagnoses were made. Of all 271 cases, CPO was diagnosed directly after delivery in 45.0 % (*n* = 122), rising to 52.7 % (*n* = 143) before the age of 1 week and to 55.7 % (*n* = 151) diagnosed children within the first 2 weeks. Eventually, 62.7 % (*n* = 170) is diagnosed within the first 30 days of age. The detection rate subsequently diminishes; for between 1 and 12 months, only 12.5 % (*n* = 34) was diagnosed. Finally, after the age of 12 months, CPO was diagnosed in 24.8 % (*n* = 67). The mean age at diagnosis was 502 days (1 year and 4 months) with an SD of 1080, median of 3 and an IQR of 342 days. It is noteworthy that the highest age at diagnosis was 26 years (patient X; grade 1 CP), an outlying case, twice the age of the penultimate outlier. When patient X is excluded, the mean age at diagnosis was 469 days (SD = 932).

**Table 2:** Age of all children at diagnosis of CPO

Age at diagnosis CPO			
	*n* (total *n* = 271)	%	Cumulative %
Post-partum	122	45.0 %	45.0 %
Post-partum–1 week	21	7.7 %	52.7 %
1–2 weeks	8	3.0 %	55.7 %
2–3 weeks	10	3.7 %	59.4 %
3–4 weeks	9	3.3 %	62.7 %
1–3 months	17	6.3 %	69.0 %
3–6 months	8	2.9 %	71.9 %
6–12 months	9	3.3 %	75.2 %
>12 months	67	24.8 %	100.0 %
≤14 days	151	55.7 %	55.7 %
>14 days	120	44.3 %	100.0 %
≤30 days	170	62.7 %	62.7 %
>30 days	101	37.3 %	100.0 %
Mean and SD (days)	502.49 and 1080.222		
Mean and SD (patient X excluded)	469.17 and 932.249		
Median and IQR (days)	3.00 and 342.00		

### Extent of CP

Of all 271 cases, Table 3 outlines that the most common type of cleft was grade 1 (27.7 %; *n* = 75), while grade 2 was less common (23.2 %; *n* = 63). Grade 3 occurred in 24.0 % (*n* = 65), and finally grade 4 occurred in 25.1 % (*n* = 68). Within the submucous group, 47 children had a bifid uvula (62.7 % of grade 1), and nine children (12.0 % of grade 1) were diagnosed with velopharyngeal insufficiency and/or hypoplasia of the palate. Though a cleft had not been detected in these eight patients, the typical CP symptoms of nasal regurgitation and hypernasality were present, and hypotonia/hypotrophy of the velar muscles had been diagnosed. Two of these eight patients were diagnosed with velo-cardio-facial syndrome (VCF). Of the patients diagnosed late (>30 days), most had a submucous cleft (66.3 %; *n* = 67), and as the severity of the cleft increased, late detection occurred less often: within 30 days, 22 grade 2 patients were diagnosed, seven grade 3 patients and five grade 4 patients. A chi-squared test revealed a highly significant association between the grade of the cleft (1–4) and the age at diagnosis (≤30 days (early)/>30 days (late); *p* = .000); as the severity of the CP increased, the chances of early diagnosis also increased. Early diagnosis occurred most frequently in children with a grade 4 CP (92.6 % of grade 4; *n* = 63), while late diagnosis was common in grade 1 patients (89.3 % of grade 1; *n* = 67). Similar results are visible when early age at diagnosis was set at ≤14 days (*p* = .000).

**Table 3:** Categorical variables of all included children with CPO

	Total	≤30 days	>30 days	≤14 days	> 14 days
	100 % (*n* = 271)	62.7 % (*n* = 170)	37.3 % (*n* = 101)	55.7 % (*n* = 151)	44.3 % (*n* = 120)
Type of cleft		*p* = 0.000		*p* = 0.000	
1. Submucous/bifid uvula	27.7 (*n* = 75)	10.7 (*n* = 8)	89.3 (*n* = 67)	8.0 (*n* = 6)	92.0 (*n* = 69)
2. Soft palate only	23.2 (*n* = 63)	65.1 (*n* = 41)	34.9 (*n* = 22)	54.0 (*n* = 34)	46.0 (*n* = 29)
3. Soft palate + partial hard palate	24.0 (*n* = 65)	89.2 (*n* = 58)	10.8 (*n* = 7)	81.5 (*n* = 53)	18.5 (*n* = 12)
4. Complete cleft (soft + hard)	25.1 (*n* = 68)	92.6 (*n* = 63)	7.4 (*n* = 5)	85.3 (*n* = 58)	14.7 (*n* = 10)
Delivery location		*p* = 0.036		*p* = 0.003	
1. Home	25.1 (*n* = 68)	63.2 (*n* = 43)	36.8 (*n* = 25)	57.4 (*n* = 39)	42.6 (*n* = 29)
2. WCH	15.1 (*n* = 41)	80.5 (*n* = 33)	19.5 (*n* = 8)	73.2 (*n* = 30)	26.8 (*n* = 11)
3. Hospital other than WCH	52.4 (*n* = 142)	60.6 (*n* = 86)	39.4 (*n* = 56)	54.9 (*n* = 78)	45.1 (*n* = 64)
4. Abroad	1.1 (*n* = 3)	33.3 (*n* = 1)	66.6 (*n* = 2)	33.3 (*n* = 1)	66.6 (*n* = 2)
5. Unknown	6.3 (*n* = 17)	41.2 (*n* = 7)	58.8 (*n* = 10)	17.6 (*n* = 3)	82.4 (*n* = 14)
Syndromic CP (CPO+)		*p* = 0.024		*p* = 0.002	
1. Yes	46.5 (*n* = 126)	69.8 (*n* = 88)	30.2 (*n* = 38)	65.9 (*n* = 83)	34.1 (*n* = 43)
2. No	53.5 (*n* = 145)	56.6 (*n* = 82)	43.4 (*n* = 63)	46.9 (*n* = 68)	53.1 (*n* = 77)
GA cut-off (weeks)					
1. Pre-term (<37)	11.1 (*n* = 30)				
2. Full-term (≥37)	81.5 (*n* = 221)				
3. Unknown	7.4 (*n* = 20)				

### Associated disorders

Pierre Robin sequence (PRS) generally consists of a triad of symptoms entailing micrognathia, glossoptosis and the resulting post-partum respiratory distress [5]. Although there currently is no global consensus on the exact definition of PRS, there is a well-known common concomitance of CP [4]. Over 80 % of children with PRS eventually displays an associated syndrome such as, e.g. Stickler syndrome or VCF (22q11) [23]. Of all 271 cases, 46.5 % (*n* = 126) was classified as having an associated disorder (CPO+). Table 4 outlines that the majority of this group was diagnosed with PRS without any associated disorders (40.5 %; *n* = 51). It is noteworthy that all 51 patients suffered from PRS with CP. Of all 126 CPO+ patients, 14.3 % (*n* = 18) was diagnosed with Stickler’s and 11.9 % (*n* = 15) with VCF (22q11). Moreover, there was a large variety of other syndromes, summarised in Table 4.

**Table 4:** Syndromic and/or congenital abnormalities of all included children with CPO

Syndromic and/or congenital abnormalities of all included children	
	CP+ Total % (*n* = 126)
PRS	40.5 % (*n* = 51)
PRS with Stickler	14.3 % (*n* = 18)
PRS with VCFS/22q11	11.9 % (*n* = 15)
PRS with Treacher-Collins	0.8 % (*n* = 1)
CPO+ without clear diagnosis/syndrome	4.0 % (*n* = 5)
CHARGE syndrome	2.4 % (*n* = 3)
Kabuki syndrome	2.4 % (*n* = 3)
Trisomy 21 18q-syndrome Van der Woude syndrome	1.6 % (*n* = 2)
4q-, 13q-, 19q-, Auriculocondylar-, Beckwith Wiedeman-, BOR-, EEC-, Klippel Feil-, Loeys-Dietz-, Moebius-, DOOR-, orodigital-facial-, SEDH-, Adams Oliver-, Apert-, Goldenhar-, Rieger-, Triple X-, Worster Drought syndrome	0.8 % (*n* = 1)
ODS type 1, partial diprosopus, inversion duplication chromosome 15, hemifascial microsomy	0.8 % (*n* = 1)

Visible in Table 3, a chi-squared test revealed a significant association between the presence of associated disorders (yes/no) and the age at diagnosis (≤30 days (early)/>30 days (late); *p* = .024); in the late diagnosis group, CPO+ was less frequently diagnosed. Similar results are visible when early age at diagnosis was set at ≤14 days (*p* < .000).

### Location of delivery

Home deliveries occurred in 25.1 % (*n* = 68) of all patients. Furthermore, 67.5 % (*n* = 183) had been born in hospital. Within the total group, 15.1 % (*n* = 41) had been born in our institution (WCH) and 52.4 % (*n* = 142) in a regional hospital (non-WCH). Moreover, for some cases, the delivery location was unknown or unclear in the medical charts (6.3 %; *n* = 17). Finally, three children had been born in a foreign country (1.1 %; *n* = 3). As outlined in Table 3, most early diagnoses (≤30 days) were made at the WCH (80.5 %; *n* = 33), followed by the location home (63.2 %; *n* = 43) and non-WCH hospitals (60.6; *n* = 86). Moreover, early diagnoses occurred relatively infrequently in unknown (41.2 %; *n* = 7) or foreign (33.3 %; *n* = 1) settings. A chi-squared test revealed a significant association between the location of delivery (home/WCH/other Dutch hospital/abroad/unknown) and the age at diagnosis (≤30 days (early)/>30 days (late); *p* = .036). Since 62.7 % (*n* = 170) of all cases were diagnosed early regardless of location, early detections occurred roughly evenly frequently in home settings (63.2 %) and non-WCH hospital settings (60.6 %). Early detection did occur more frequently at the WCH (80.5 %). Similar results are visible when “early” was defined as ≤14 days (*p* < .003). Conversely, when all hospital births were combined (WCH + non-WCH births) and “early” was defined as ≤30 days, there was no longer a significant association between delivery location and age at diagnosis (*p =* .179). With combined hospital births and early defined as ≤14 days, a significant association remained visible (*p* = .009); early detection then occurred most often in hospitals (59.0 %; *n* = 108) and homes (57.4 %; *n* = 39), and fairly infrequently in unknown (17.6 %; *n* = 3) and foreign (33.3 %; *n* = 2) settings. Another chi-squared test revealed that there was also a significant relation between the presence of associated disorders (CPO+ yes/no) and the location of delivery (*p =* .038); syndromic diagnoses were much more frequent at the WCH (63.4 % of their cases) as compared to other hospitals (42.3 %), but in home deliveries, there was an even spread between non- and syndromic diagnoses (50 vs 50 %). Additionally, there were more CPO+ diagnoses (66.7 %; *n* = 2) abroad. When all hospital births were combined, there was no longer a significant association between delivery location and associated disorders (*p =* .217).

### Gestational age

Of all 271 cases, premature births (<37 weeks) occurred in 11.1 % (*n* = 30). An independent sample *t* test did not reveal any significant differences between age at diagnosis (≤30 days (early)/>30 days (late)) and the continuous GA variable (days) (*p* = .933). Similar results were found when early was defined as ≤14 days (*p* = .529).

Of the children born prematurely (11.1 %; *n* = 30), only ten children were diagnosed late (>30 days; 33.3 %). Of these ten late-diagnosed children, 60.0 % (*n* = 6) had a grade 1 CP, 20.0 % (*n* = 2) had a grade 2 CP and 20.0 % (*n* = 2) had a grade 3 CP. There were no premature children with a late-diagnosed grade 4 CP (*p* = .001). With “late” defined as >14 days, results were similar: in the early group, there were 6 grade 1 patients (54.5 %), 2 grade 2 patients (33.3 %), 2 grade 3 patients (33.3 %) and 1 grade 4 patient (11.1 %; *p =* .003). Finally, of all included children with CPO, 7.4 % (*n* = 20) had an unknown GA, because the information could not be retrieved from the (relevant) medical records.

### Survival analyses

**Fig. 2:**
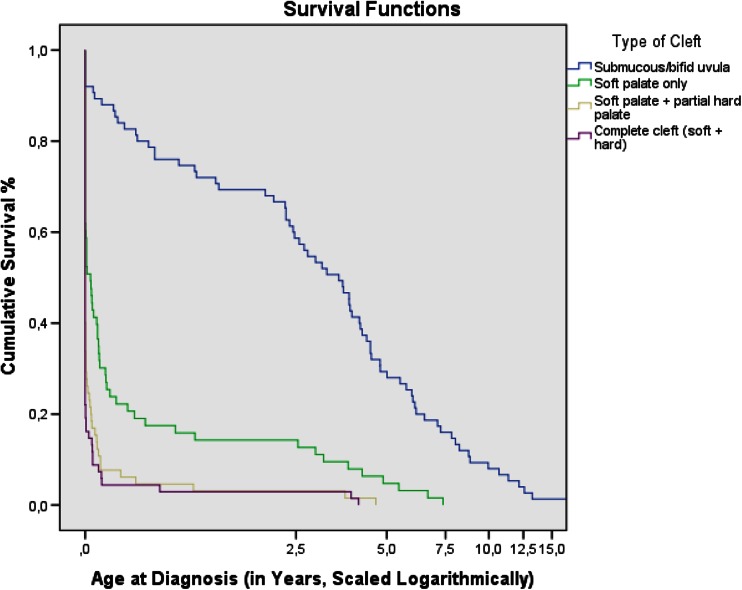
Survival function of age at diagnosis and the 4 cleft grades

In addition to the previous chi-squared analyses, several Kaplan-Meier survival analyses were conducted to compare (log rank test) the age at diagnosis with the categorical variables, resulting in Table 5. First, for cleft type, grade 1 patients had a much higher median age at diagnosis (1282 days) than grade 2 patients (12 days), who, in turn, had a higher median age than grades 3 and 4 patients (0 and 0 days, respectively; *p* < .001). Using a Bonferroni correction at the *p* < .0125 level, a pairwise log rank comparison revealed significant differences (*p* < .001) in all grade interactions (1 vs 2, 1 vs 3, etc.), except between grades 2 vs 4 (*p* = .276). These differences have been further visualized in Fig. 2. Second, for delivery location, patients born abroad had a much higher median age at diagnosis (1407 days) than patients born at home (4 days), and these home-borns in turn were diagnosed later than patients born in a hospital other than the WCH (2 days), which, in turn, was later than children born at the WCH (0 days; *p* < .021). Using a Bonferroni correction at the *p* < .01 level, a pairwise log rank comparison revealed that none of the possible pair interactions were significant, except between WCH versus unknown (*p* = .006). Conversely, when all hospital births were combined (WCH + non-WCH), the association between location of delivery and age at diagnosis did not remain significant (*p* = .263). Third, for associated disorders, syndromic patients had lower median age at diagnosis (0 day) compared to the non-syndromic patients (18 days; *p* = .007). Finally, cut-off gestational age (pre-term/full-term/unknown) did not significantly relate to age at diagnosis (*p* = .107).

**Table 5:** Kaplan-Meier survival analyses of age at diagnosis (days)

	Median (days)			
			95 % CI	
	Estimate	SE	Lower bound	Upper bound
Type of cleft	Log rank: *χ* ^2^ (3) = 152.685; *p* = .000			
1. Submucous/bifid uvula	1282.0	197.725	894.5	1669.5
2. Soft palate only	12.0	5.732	0.8	23.2
3. Soft palate + partial hard palate	0.0	.	.	.
4. Complete cleft (soft + hard)	0.0	.	.	.
Delivery location	Log rank: *χ* ^2^ (4) = 11.610; *p* = .021			
1. Home	4.0	5.497	0.0	14.8
2. WCH	0.0	.	.	.
3. Hospital other than WCH	2.0	.	.	.
4. Abroad	1407.0	1148.811	0.0	3658.7
5. Unknown	167.0	122.107	0.0	406.3
Syndromic CP (CPO+)	Log rank: *χ* ^2^ (1) = 7.400; *p* = .007			
1. Yes	0.0	.	.	.
2. No	18.0	6.397	5.5	30.5
GA cut-off (weeks)	Log rank: *χ* ^2^ (2) = 4.479; *p* = .107			
1. Pre-term (<37)	0.0	.	.	.
2. Full-term (≥37)	2.0	.	.	.
3. Unknown	167.0	146.462	0.0	454.1

### Reasons for referral in the late diagnosis group

The most frequently reported symptoms of children diagnosed late included feeding difficulties, hearing and speech/language problems (development). Most often problems had been present for a long time due to the unrecognised CPO. Nasal regurgitation was most prevalent in children under the age of 1; older children (>2 years old) frequently presented problems regarding the development of speech, despite intensive speech therapy.

Finally, a number of children had only presented with symptoms of late speech development, and during the subsequent speech therapy, some speech therapists were able to recognise the cleft soon after the treatment had started, while others had not even detected the cleft in a situation where they had been working with the patient for years.

## Discussion

The present study reveals that a large proportion (37.3 %) of all children included were diagnosed with CPO more than 30 days after birth, even though the national guideline suggests that every live-born child with an OC should preferably be seen by the regional CPT within 2 weeks after birth [8]. In our clinic, most children were seen between 2 and 4 weeks after birth; very rarely were they examined after the age of 4 weeks. In such cases, this was due to extraneous practical/logistic reasons such as holidays and parental illness. Currently, no data exists about the exact day a cleft is first suspected by either parents or health care practitioners; such diagnoses are only registered in the DCR when children first visit a CPT. In this study, since children are rarely seen later than 30 days, a clinically relevant cut-off point of 30 days was chosen in order to differ between early and late diagnoses of CPO. However, our findings remained valid when a cut-off point of 14 days was chosen. Additionally, to overcome the limitations of these dichotomous statistical methods, Kaplan-Meier analyses were added, again validating our findings.

Results are in concordance with the existing literature. Habel et al. demonstrate that CP is not detected on the first day after birth in 28 % of all cases, and on the second day after birth in 20 % of all cases [12]. Late diagnosis is especially common in the submucous-grade CP [12]. Furthermore, a Swedish study reports that none of its 39 included children with CPO had been diagnosed during prenatal screening [3]. In contrast, almost all children with CL/P are diagnosed before the age of 1 year [2, 3]. Moreover, when CPO is diagnosed after the age of 1 year, this most often occurs between the ages of 3 and 6 years [2, 3].

A significant association between the presence of associated disorders and the age at diagnosis (*p* = .024 with cut-off at 30 days; *p* = .000 with cut-off at 14 days) was revealed, a finding which remained valid when analysed with Kaplan-Meier statistics (*p* = .007). In the late diagnosis group, CPO+ occurred less frequently. This result either suggests that CPO is more difficult to detect without any concomitant abnormalities, or that children with concomitant abnormalities are better examined in general. Since children with associated disorders are already under medical supervision, it is conceivable that the high exposure to medical professionals directly leads to an increased chance for detecting CPO at an early age. However, the results also demonstrate that even children with an associated malformation could be diagnosed late (CPO+ in late group, *n* = 38). Hence, health care professionals confronted with children with an associated disorder in conjunction with nasal regurgitation, other feeding difficulties or symptoms as described above should be aware of a possible underlying CP.

Second, not in line with expectations, there were conflicting results on associations between the location of delivery and the age at diagnosis (early/late) when all hospital births were combined; this association was significant with a cut-off of 14 days (*p = .*009), but not significant with a cut-off of 30 days (*p* = .179). We had expected more early diagnoses in a hospital setting, as was reflected in the results when hospital location was diversified to non-WCH and WCH. When diversified, there was a significant association between location and detection age (*p* = .036); more early diagnoses were made at the WCH, as compared to other locations. However, since the WCH is a specialised children’s hospital, this finding was not unexpected. More children with associated disorders were born at the WCH compared to other locations (*p* = .038). Furthermore, the survival analyses also revealed a significant association between location of delivery and age at diagnosis (*p* = .021), though a post hoc test only showed a significant difference between patients born at the WCH and children born at an unknown location. When hospitals were combined, this association did not remain significant (*p* = .263). We suggest that the reason for the non-significant difference between delivery location and detection age could be that midwives generally are well capable of detecting CPO, regardless of the place in which they work (non-medical/medical environment). Unlike in foreign countries, in the Netherlands, 25.0 % of all infants are delivered at home, under supervision of a midwife, while 75.0 % of the deliveries are performed in a hospitalised setting [13].

Third, there was no significant association between age at diagnosis and GA (*p* = .993 with cut-off at 30 days; *p =* .529 with cut-off at 14 days; *p =* .107 with Kaplan-Meier survival analysis), even though we had expected that post-partum examinations of pre-term babies, occurring in specialised neonatal environments, would lead to higher detection rates.

Finally, when focusing on the grade of the cleft, the present study reports a highly significant association between grade (1–4) and the age at diagnosis (early/late), regardless of cut-off point (*p* = .000); as the severity of the CP increases, the chances of early diagnosis also increase. Similar results were seen in the survival analysis comparing cleft grade and continuous age at diagnosis (*p* = .000). This is especially relevant for the submucous CPs (grade 1), in which the cleft of the levator veli palatini muscle is often invisible, though a cleft/bifid uvula may be observed. In line with this, Ha et al. report that 98 % of their patients with a CPO grade 1 presented with a bifid uvula [11]. Furthermore, symptoms to be aware of are feeding difficulties combined with nasal regurgitation and ear problems such as conductive hearing loss and persistent otitis media. At a later stage in the child’s development, hypernasality and articulation problems should alert speech pathologists and pediatric otolaryngologists, prompting them to thoroughly inspect the mouth in search of CPO [11, 24]. Additionally, the soft palate often fails to elevate sufficiently at times of crying and feeding, or at a later stage during speech development, causing open nasality. Our findings suggest that during post-partum examinations, the palate is often examined inadequately. This signifies that health professionals should be made more aware of the various symptoms of CPO, specifically those of the submucous type (grade 1). Therefore, increased and more widespread knowledge about CPO and its symptoms, in conjunction with the implementation of a standardised manual palpation of the palate during the initial physical examination of a newly born infant, are likely to lead to more successful detections and diagnoses.

It is imperative to realise that the results of the present study also indicate that other grade CPs (2–4) are also diagnosed late (>30 days; grade 2, *n* = 22, 34.9 %; grade 3, *n* = 7, 10.8 %; grade 4, *n* = 5, 7.4 %). This is surprising, since these clefts are visible, and hence easier to detect. These late diagnoses stress the need for proper physical post-partum examinations. We propose that early detection of submucous clefts can be improved, though this will likely be a challenge due to its detection difficulty. In contrast, we believe it should be possible to improve our rate of late diagnoses of grades 2–4 CPs, subsequently preventing the associated health problems. This is supported by Habel et al. [12], who report that late diagnoses occur more often with narrow V-shaped clefts, as opposed to broad U-shaped clefts, which are detected earlier [12]. Furthermore, they state that clefts of the soft palate are diagnosed later than clefts of the hard palate. As the underlying cause, they suggest that a lack of proper visual inspection lies at the root of late detection.

For optimal management of the oral cleft, it is imperative that CPO is diagnosed as early as possible. Likewise, parents can only be properly informed about and assisted with their infant’s CPO-induced problems after a diagnosis has been made. Parents in this study reported that they had sometimes been caught in a situation in which they kept being referred without getting the necessary information or treatment. A dire situation for parents arose; mothers reported that they had the instinctive feeling that something was wrong with their child, which consequently led them to discover the CPO on her own. Health professionals were subsequently often reluctant to refer the child to the hospital, and parent’s persistent demand for referral was often necessary. Undiagnosed CPO leads to major disadvantages in the child’s development of feeding (nasal regurgitation) and speech (open nasality, phonological problems, communication problems and voice problems) [7, 16]. Therefore, improving the post-partum detection rate of CPO is necessary in order to prevent problems at later age. Paediatricians, midwives, ENT surgeons and speech therapists should be made more aware of CPO and its symptoms: when feeding difficulties and speech/hearing/developmental problems do not improve despite “proper” treatment, CPO should be considered as the underlying cause. Furthermore, Habel et al*.* [12] propose that both manual (digital) as well as visual inspections should become a standardised part of all post-partum physical examinations. They continue to describe the potential pitfalls for both manual and visual examinations: the interpretation of sensory input during the manual palpation of the palate can be difficult, especially when the performing health professionals are inexperienced. Moreover, when a cleft is present, it is possible to touch the vomer in the cleft, which can be misinterpreted as a closed palate. Third, they describe that an incomplete depression of the tongue during visual inspection could lead to an inadequate view of the mouth and palate. They state that this is highly likely one of the main reasons why visual detection often fails.

In conclusion, properly performed visual inspections seem essential, and a combination of both manual and visual examination types will likely lead to improved detection rates. This is in line with the World Health Organization (WHO), who advise visual examinations in developing countries [26]. We propose that western-oriented health professionals should also follow this advice.

Since all data in this study have been reviewed retrospectively from actual hospital cases, some degree of confounding may be present. Moreover, the Dutch Cleft Registry was used as a basis for data analysis, and even though the data in this study was derived from one of the largest cleft teams in the country, it is possible that the incidence per region might differ. Finally, over the period of 1997–2014, a degree of “secular change” has taken place: the standard of care has become slightly different (e.g. pre-natal screening), possibly leading to differing ages at diagnosis, which might affect statistical analyses. However, since multiple statistical methods were used in conjunction, we believe that our findings remain strong. However, more studies are needed to further corroborate these findings. In these future studies, the long-term consequences of a late diagnosis should also be examined.

## Conclusion

The present study has demonstrated that in the referral area of our cleft team in the Netherlands, 37.3 % of all children with CPO are diagnosed relatively late (>30 days after birth), findings which remain valid when a cut-off point of 14 days is used (44.3 % late). Moreover, when the data was analysed using Kaplan-Meier survival statistics, similar results were found: the extent of the cleft is a determining factor for early successful diagnosis. It is suggested that midwives and paediatricians should perform more thorough intra-oral investigations of all new-borns, including both a manual palpation, as well as a visual inspection of the palate. They should be made aware of the pitfalls of these inspections as well. Furthermore, these investigations should become a standardised part of all post-partum physical examinations. Moreover, it is suggested that midwives, paediatricians and paediatric otolaryngologists should be made more aware of the frequent symptoms accompanying CPO: feeding difficulties in new-borns, including nasal regurgitation and/or repetitive hearing problems requiring middle ear tubes. Finally, patients needing prolonged speech therapy, especially those presenting with nasal speech and compensatory articulation, should alert speech pathologists about the possibility of CPO.

## Abbreviations

CI: Confidence interval
CL: Cleft lip
CP: Cleft palate
CPO: Cleft palate only
CPO+: Cleft palate only with an associated disorder
CL/P: Cleft lip with palate
CPT: Cleft palate team
DCR: Dutch cleft registry
*df*: Degrees of freedom
GA: Gestational age
IQR: Interquartile range
NVSCA: National Dutch Cleft Registry
PRS: Pierre Robin sequence
SD: Standard deviation
SE: Standard error
VCF: Velo-cardio-facial syndrome
WCH: Wilhelmina Children’s Hospital
WHO: World Health Organization
